# Deep Dissecting Hematoma Requiring Surgical Removal During Veno-Venous Extracorporeal Membrane Oxygenation Management: A Case Report

**DOI:** 10.7759/cureus.65976

**Published:** 2024-08-01

**Authors:** Taichi Kotani, Yusuke Naito, Shota Sonobe, Junji Egawa, Masahiko Kawaguchi

**Affiliations:** 1 Department of Anesthesiology, Nara Medical University, Kashihara, Nara, JPN

**Keywords:** dermatoporosis, steroids, covid-19, vv-ecmo, anticoagulant therapy, deep dissecting hematoma

## Abstract

Deep dissecting hematoma (DDH) is a disease in which minor trauma leads to the formation of an extensive hematoma. If left untreated, this can result in significant skin necrosis. Therefore, early treatment following a precise diagnosis is essential. However, the complexity of the disease may require differentiating it from soft tissue infections. A 58-year-old man with severe coronavirus disease 2019 (COVID-19) pneumonia developed skin complications such as purpura and blisters on his right upper extremity while undergoing veno-venous extracorporeal membrane oxygenation (VV-ECMO). We initially suspected a soft tissue infection or venous perfusion defect caused by the VV-ECMO cannula; however, these conditions were not observed. After making an exploratory incision, we diagnosed the patient with DDH and performed hematoma removal and skin grafting. The initial symptoms of DDH include erythema, swelling, and pain. It is important to differentiate DDH from soft tissue infections, especially necrotizing fasciitis, which is a more urgent condition. Because a surgical incision is necessary for the diagnosis and treatment of DDH, we do not hesitate to perform an exploratory incision to prevent skin necrosis, thereby contributing to early healing.

## Introduction

Deep dissecting hematoma (DDH) is a disease in which minor trauma causes an extensive hematoma, which may lead to extensive skin necrosis due to delayed intervention [1,2]. DDH is more common in elderly patients with fragile skin. The mean age of onset is 81.7 years, and the most common site of onset is reported to be the lower extremities [1].

The term "dermatoporosis" describes the skin condition in which atrophy and weakening of the skin and subcutaneous tissue easily produce purpura, epidermal peeling, and subcutaneous hematoma under minor external forces, analogous to osteoporosis [3]. DDH, the most severe form of dermatoporosis, is often accompanied by localized erythema and heat and is frequently misdiagnosed as a soft tissue infection [1].

Although the differentiation method between the two diseases is a test puncture or exploratory incision [2], we sometimes hesitate to perform surgical interventions while a patient is on anticoagulation therapy. Herein, we report the case of a patient with severe coronavirus disease 2019 (COVID-19) pneumonia who was diagnosed with DDH by surgical incision during veno-venous extracorporeal membrane oxygenation (VV-ECMO).

## Case presentation

A 58-year-old male (height: 168 cm; body weight: 68 kg; body mass index 24.1 kg/m²) diagnosed with severe COVID-19 pneumonia was admitted to our intensive care unit. He had a medical history of diabetes (hemoglobin A1c: 11%) since the age of 50 years. Prior to hospital admission, he was taking dapagliflozin propylene glycolate hydrate 5 mg and vildagliptin 100 mg daily. He had smoked 90 cigarettes per day for 38 years to date. Because his oxygenation capacity was very low (SpO2 was 88% with 10 L/min of oxygen via reservoir mask), we immediately intubated him and started invasive ventilation. The mechanical ventilator settings were as follows: pressure control ventilation (PCV) mode with an FiO2 of 0.9, a positive end-expiratory pressure (PEEP) of 12 cmH2O, and a PCV pressure of 18 cmH2O (PaO2 of 68.2 mmHg and PaCO2 of 42.9 mmHg). The PaO2/FiO2 ratio was 75.8, and his Murray score was 3.5 [4]. We decided to apply VV-ECMO because his oxygenation level continued to deteriorate despite high PEEP and FiO2.

**Figure 1:**
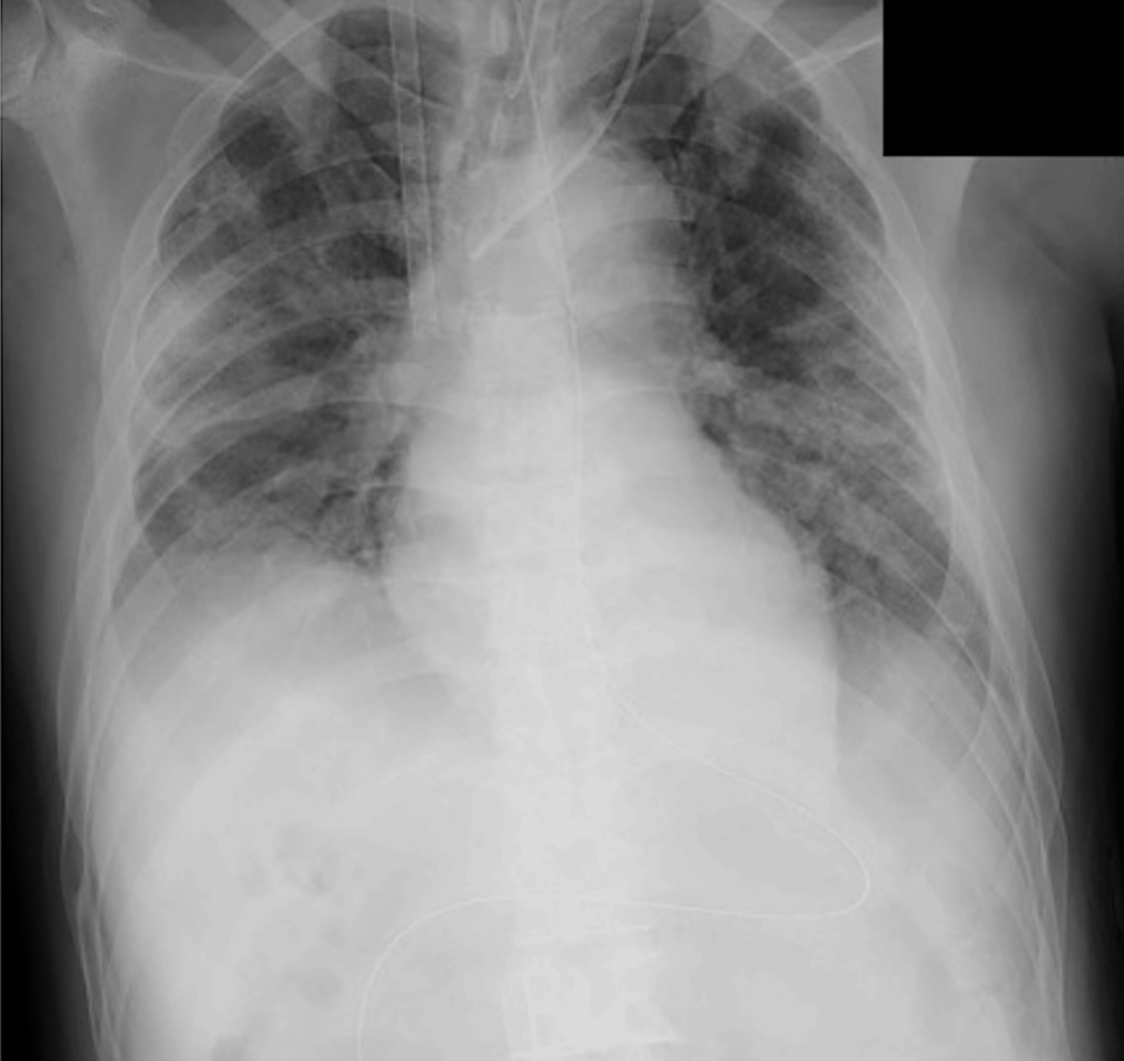
Chest radiography after cannulation

We performed cannulation via the right femoral (23 French cannula)-right internal jugular (21 French cannula) vein approach under fluoroscopic guidance (Figure 1). 

After VV-ECMO application, we changed the PCV setting to maintain a PCV pressure of less than 10 cmH2O and a PEEP of less than 10 cmH2O and adjusted heparin anticoagulation to maintain an activated coagulation time of 180-220 seconds. Remdesivir and dexamethasone were then administered. We sedated him with propofol, dexmedetomidine, and fentanyl but maintained him at a Richmond Agitation-Sedation Scale score of -1 to ensure that he would not be over-sedated during the daytime.

**Figure 2:**
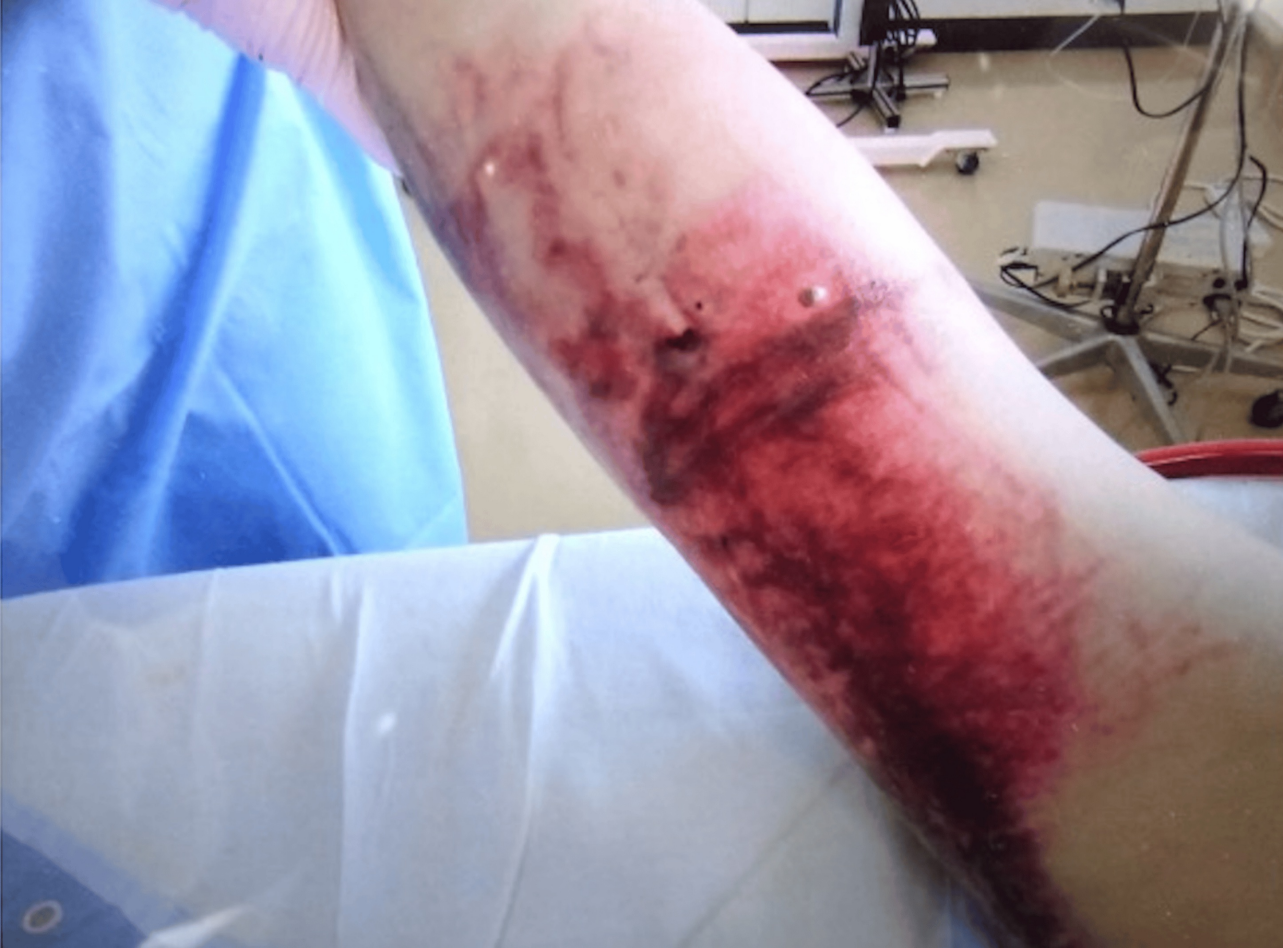
The patient’s right upper extremity on the seventh day, enlargement of purpura

On the fourth day, the intravenous catheter placed in the right median antebrachial vein was removed because of extravascular leakage. On the same day, we observed edematous changes, a subcutaneous hematoma, and blister formation at the site of leakage (Figure 2).

**Figure 3:**
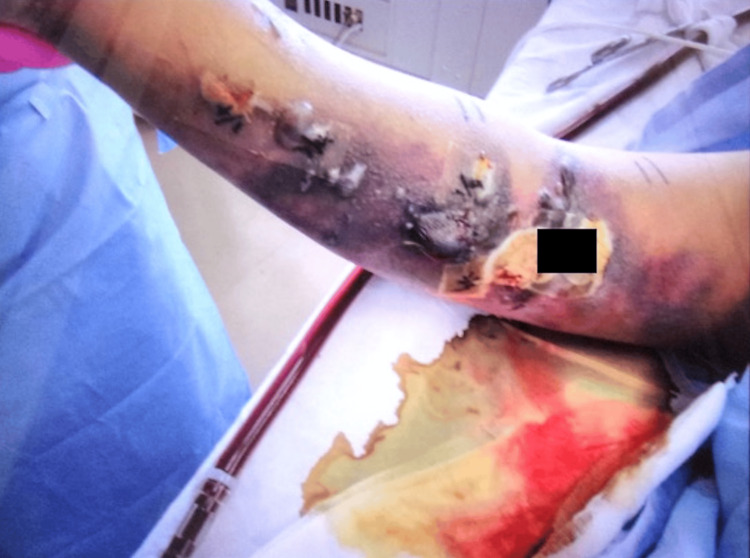
The patient’s right upper extremity on the 12th day, multiple blisters and erosions in addition to purpura

The patient experienced a severe feeling of tightness and spontaneous pain, and we suspected cellulitis or necrotizing fasciitis; however, no burning sensation was observed. In this case, we did not actively suspect the development of cellulitis or necrotizing fasciitis at this point because of the absence of a burning sensation that is commonly observed in the early stages of soft tissue infections. Concurrently, the patient was diagnosed with ventilator-associated pneumonia and started on vancomycin because of elevated C-reactive protein and Gram-positive cocci in the sputum. Although his respiratory failure improved, the skin lesions on the right upper arm worsened. On the 12th day, we performed a test puncture of the blister owing to the presence of multiple blisters and erosions (Figure 3).

**Figure 4:**
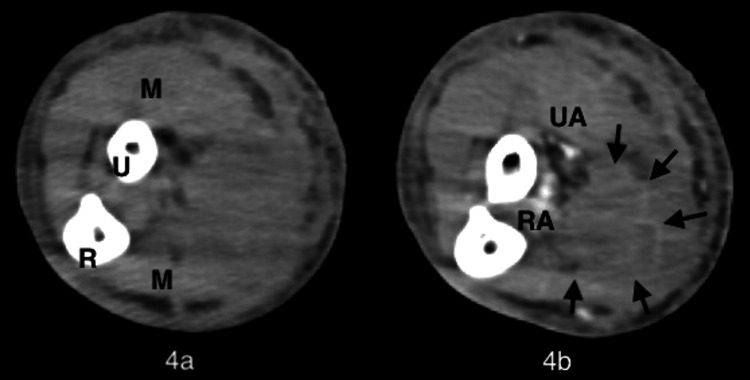
Computed tomography (CT) of the right upper extremity (a) Plane CT; (b) contrast-enhanced CT. The arterial and muscular contrast effects are good. The area surrounded by arrows shows poor internal contrast and partial external contrast, suggesting a hematoma. Arrows, hematoma; M, muscles; R, radius; RA, radial artery; U, ulna; UA, ulnar artery.

However, because the blisters had no pus drainage and the tissue culture was negative (normal flora), we assumed that there was no soft tissue infection. We suspected that the right internal jugular vein cannula of the VV-ECMO caused impaired venous perfusion of the right upper extremity and the development of compartment syndrome. On the same day, we weaned off VV-ECMO and removed the right venous cannula because the pneumonia had improved. However, there was no change in the lesions on the right upper extremity. Contrast-enhanced computed tomography (CT) of the right upper extremity revealed a hematoma in the subcutaneous tissue (Figure 4).

**Figure 5:**
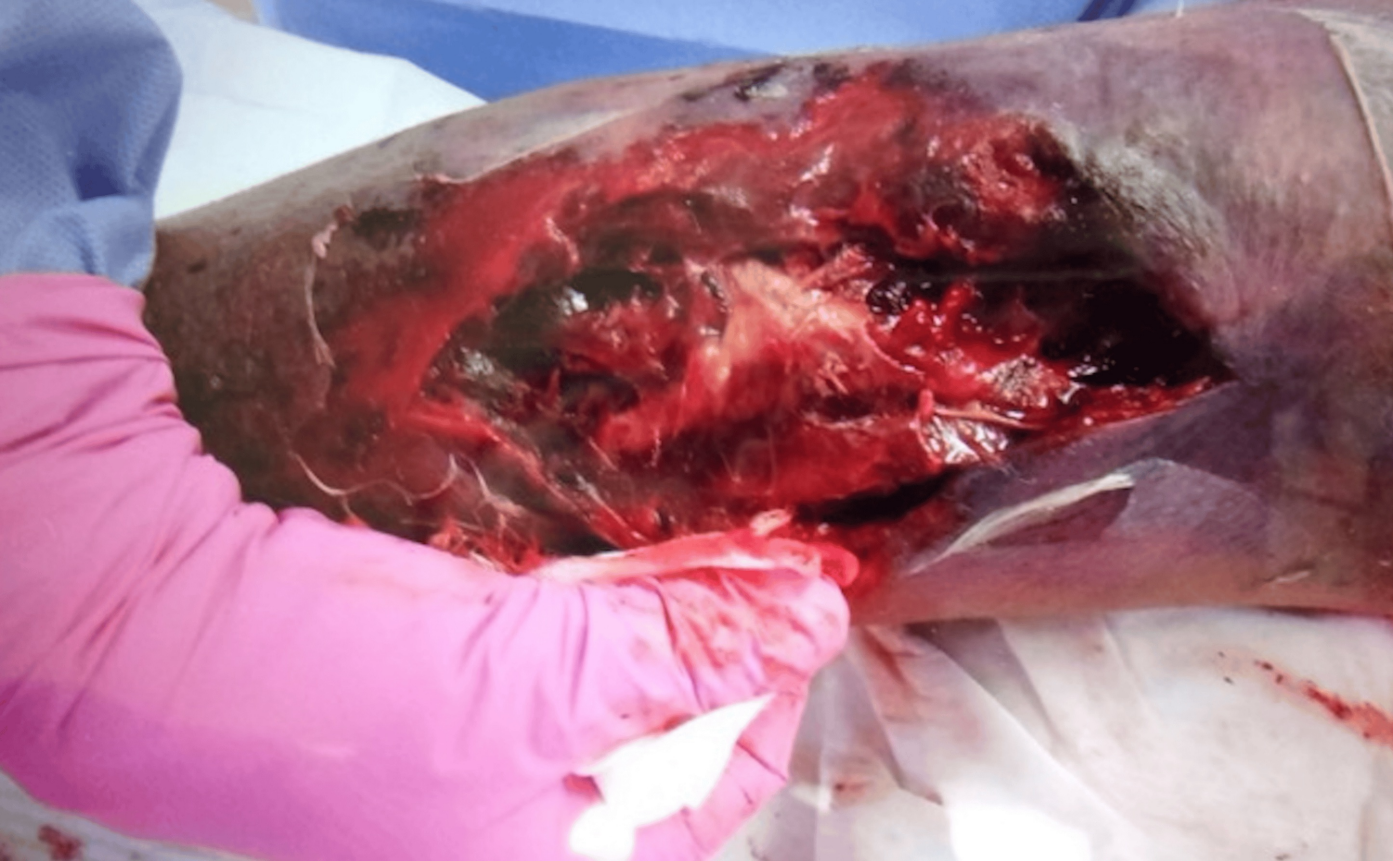
An exploratory incision of the right upper extremity There is hematoma formation within the subcutaneous tissue and dark red ischemic fatty tissue.

An exploratory incision was made at the site. We found dark red ischemic fatty tissue and a hematoma between the subcutaneous fat layer and fascia, but there was no abscess (Figure 5).

**Figure 6:**
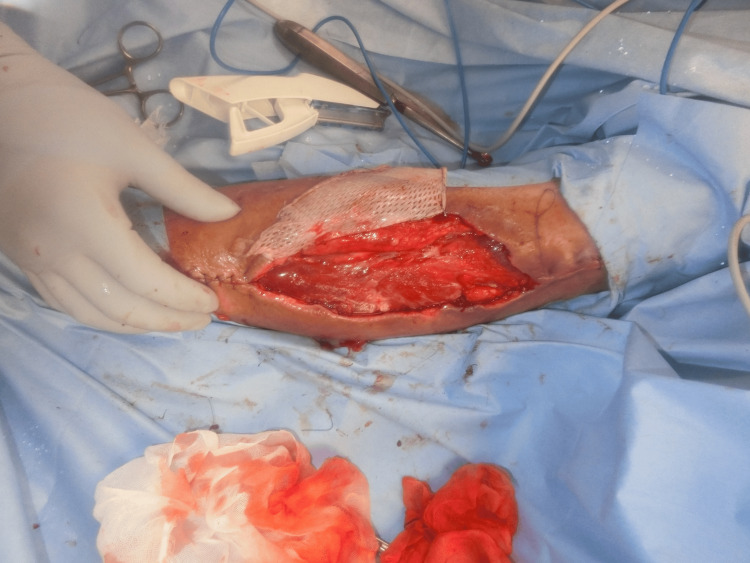
After hematoma removal, at the time of skin graft surgery

We diagnosed the patient with DDH and performed a hematoma removal. The hematoma compressed the surrounding vascular perforating branches and caused skin necrosis; however, no muscles were damaged. After the removal of the necrotic area and negative-pressure wound therapy, the patient underwent skin grafting on the 25th day (Figure 6).

We treated him during his hospitalization with the following agents: anticoagulation for VV-ECMO was administered from the first day to the 12th day. Heparin was administered continuously at approximately 500 units/hour to 1200 units/hour. After weaning off VV-ECMO, the dose was reduced to 500 units/hour and was discontinued on the 24th day before the skin grafting procedure. Changes in platelet counts, activated partial thromboplastin time, and prothrombin time (% and international normalized ratio) during anticoagulation are listed in Table 1.

**Table 1:** Changes in platelets, APTT, and PT (% and INR) during anticoagulation APTT, activated partial thromboplastin time; PT, prothrombin time: INR, international normalized ratio.

Day	1	2	3	4	5	6	7	8	9	10	11	12	13	14	15	16	17	18	19	20	21	22	23	24
Platelets (× 10^3^/µL)	391	389	310	301	321	263	236	260	266	232	195	171	163	198	210	252	267	310	316	333	326	340	347	435
APTT (s)	34	70.6	66.8	74	66.8	46.2	48.3	58.2	57.1	63.9	64.9	64.3	65.3	38	36.6	35.1	34.2	32.8	33.7	32.2	37.3	36.4	36.6	42.8
PT-%	31	57	68	68	68	77	69	70	76	80	83	79	89	89	82	91	91	99	104	107	99	98	106	107
PT-INR	1.4	1.46	1.29	1.28	1.29	1.19	1.28	1.26	1.2	1.15	1.13	1.16	1.02	1.07	1.13	1.06	1.06	1	0.98	0.96	1	1.01	0.97	0.96

For the treatment of COVID-19 pneumonia, the patient received remdesivir 100 mg and dexamethasone 6.6 mg daily from the first day to the seventh day. From the seventh to the 21st day, vancomycin 2 g daily was administered for ventilator-associated pneumonia. For sedation during ventilator management, we administered propofol 100 mg/hour, dexmedetomidine 0.7 micrograms/kg/hour, and fentanyl 100 µg/hour, respectively. We extubated the patient on the 21st day when we diagnosed that his ventilator-associated pneumonia had resolved. All sedative medications were terminated with extubation.

**Figure 7:**
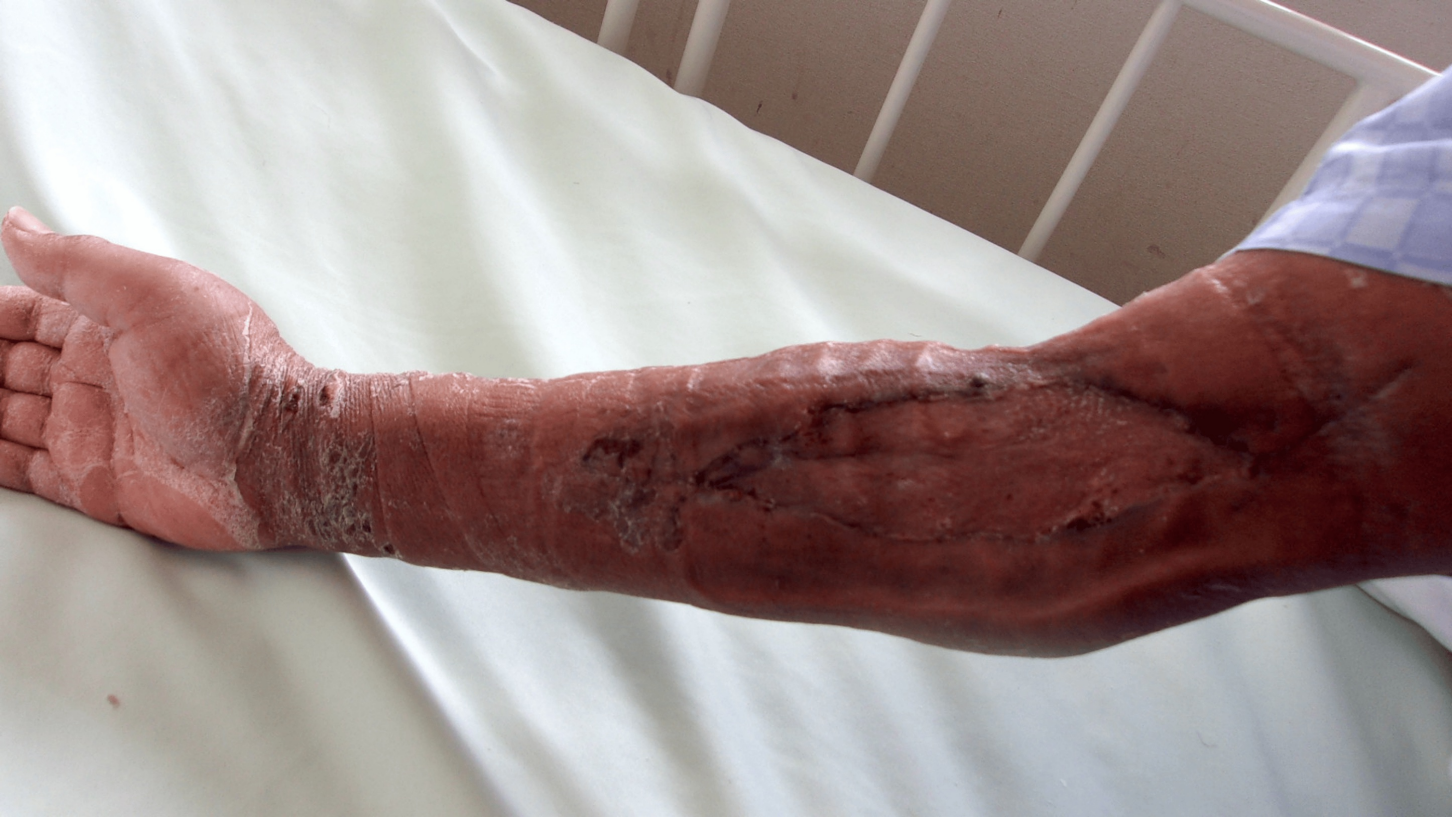
Implanted skin area at discharge

**Figure 8:**
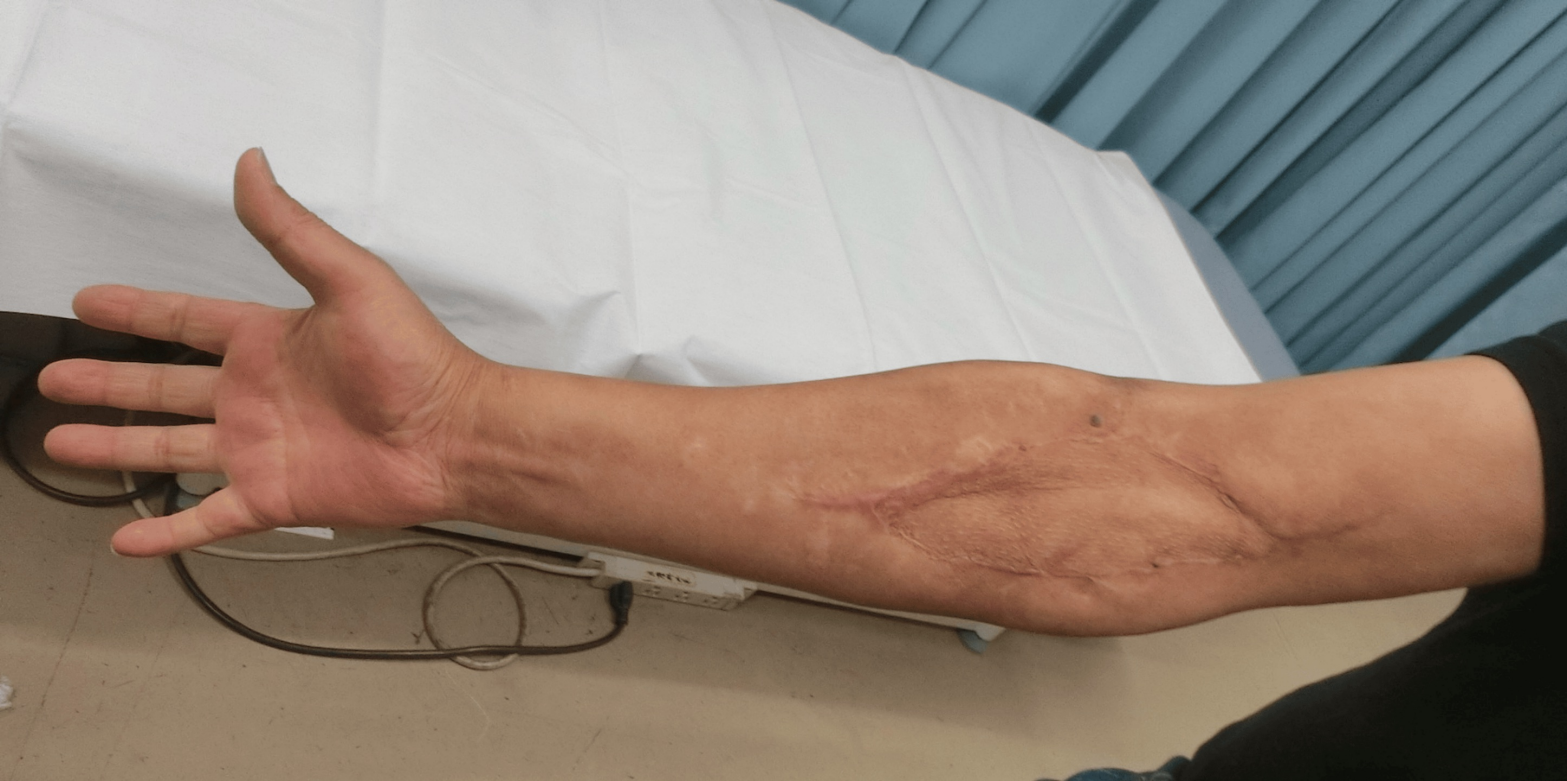
Implanted skin area at one year after discharge

He was discharged from our hospital owing to respiratory improvement on the 39th day. At discharge, the implantation was well established (Figure 7); one year later, no abnormalities at the implantation site (Figure 8) and no neurological sequelae developed.

## Discussion

Herein, we present a case of DDH during VV-ECMO in a patient with severe COVID-19 pneumonia. We presumed that his skin lesions originated first from a soft tissue infection and then from impaired venous perfusion; however, this was not the case.

Kaya and Saurat proposed the concept of "dermatoporosis" to explain skin fragility and functional decline in older patients [3]. Dermatoporosis is classified into four stages based on clinical symptoms, and DDH is classified as stage 4 [3,5].

DDH is a skin condition in which a mild impact causes avulsion between the subcutaneous fatty tissue and fascia, forming a large hematoma. Dermatoporosis is characterized by marked atrophy and thinning of the epidermis and dermis compared to normal skin, shallow exposure of blood vessels in the subcutaneous fatty tissue, and a tendency to bleed even with minor trauma. A subcutaneous hematoma resulting from this mechanism is defined as DDH. DDH is a disease that predominantly affects the elderly, but other risk factors, such as oral anticoagulants and diabetes mellitus, have also been reported (Table 2) [2]. Initial skin manifestations of DDH include erythema and swelling. In severe cases with an enlarged hematoma, internal compression may later cause considerable skin necrosis and form intractable ulcers. It is difficult to determine the severity of the disease from the initial imaging examination alone. Therefore, it is necessary to observe the clinical findings over time and perform another imaging examination if there is any exacerbation. When the hematoma increases rapidly, the pressure in the subcutaneous tissue increases, causing severe pain. Although complaints of pain vary from person to person, it is essential to monitor the patient's condition, including changes in complaints. Moreover, if removal is delayed, the hematoma compresses the surrounding perforating branches of arteries, resulting in extensive skin necrosis [1,2]. It is an urgent condition, and early diagnosis and treatment are essential [6].

**Table 2:** Patient background with deep dissecting hematoma

Patient background	Value
Anticoagulant and antiplatelet medication	50%
Diabetes mellitus	18%
Steroids long-term internal use	12%

However, it is difficult to diagnose DDH based on skin lesions alone. In this case, we first suspected the development of a soft tissue infection based on symptoms such as blister formation and spontaneous pain. The initial symptoms of DDH include erythema, swelling, and pain. It is important to differentiate DDH from soft tissue infections, especially necrotizing fasciitis, a more urgent condition [1]. Kaya et al. reported that of 34 patients diagnosed with DDH, 14 were diagnosed with soft tissue infection at the initial visit, and eight were treated with antimicrobials [2].

The common denominator in the diagnosis and treatment of DDH, soft tissue infections such as necrotizing fasciitis, and compartment syndrome is a prompt exploratory incision. Delayed treatment of either disease can lead to necrosis of not only the skin but also fascia and muscle, resulting in permanent motor dysfunction [6-8]. DDH took, on average, approximately two weeks or more from the onset of skin lesions to diagnosis [2]. We weaned the patient off VV-ECMO and discontinued heparin on the 12th day, which allowed us to make an exploratory incision and diagnose DDH. Fortunately, there was no evidence of necrosis, but we could not rule out the possibility that the exploratory incision was delayed because of concerns regarding heparin-induced hypocoagulability. On the seventh day, the skin lesions may have necessitated an exploratory incision.

We also suspected that the right internal jugular vein cannula of the VV-ECMO caused impaired venous perfusion and the development of compartment syndrome. Classically, the presentation of compartment syndrome has been remembered by “The Five P’s”: pain, pulselessness, paresthesia, paralysis, and pallor [7]. In this case, the patient experienced pain but none of the other four signs. Ultrasonography revealed blood flow in the brachial, radial, and ulnar arteries, which were considered negative for extensive ischemic involvement. Furthermore, there have been no reports of impaired blood flow in the upper extremities due to the VV-ECMO cannula, and we were skeptical about its pathogenesis.

There have been no reports of DDH during the treatment of severe COVID-19 pneumonia or the use of VV-ECMO. Skin vulnerability from heparin and corticosteroids used for COVID-19 treatment was possibly responsible for the development of DDH in our case, who was relatively young and unlikely to develop dermatoporosis.

Early skin incision allowed this patient to be discharged without neurological sequelae. Delayed intervention in the treatment of DDH can lead to muscle necrosis due to hematoma compression and the development of neurological sequelae. DDH is a complex disease to diagnose based on cutaneous symptoms. Although contrast-enhanced CT may aid in the diagnosis of DDH, only early diagnostic therapeutic intervention can confirm DDH.

## Conclusions

We encountered a case of DDH requiring surgical removal during VV-ECMO management. DDH is a complex disease to diagnose at an early stage. However, clinicians should take early imaging, especially contrast-enhanced CT, and should not hesitate to perform an exploratory incision to prevent skin necrosis, which will contribute to early healing.
